# Monte Carlo analysis of light fluence rate distribution in pleural photodynamic therapy: a study of geometric and optical property effects on treatment delivery

**DOI:** 10.1117/1.JBO.31.1.018001

**Published:** 2026-01-05

**Authors:** Hongjing Sun, Madelyn Johnson, Dennis Sourvanos, Timothy C. Zhu

**Affiliations:** a University of Pennsylvania, Perelman Center for Advanced Medicine, Department of Radiation Oncology, Philadelphia, Pennsylvania, United States; b University of Pennsylvania, Department of Bioengineering, Philadelphia, Pennsylvania, United States; c University of Pennsylvania, School of Dental Medicine, Department of Periodontics, Philadelphia, Pennsylvania, United States

**Keywords:** pleural photodynamic therapy, Monte Carlo, light delivery

## Abstract

**Significance:**

Pleural photodynamic therapy (PDT) faces significant dosimetry challenges due to complex light distribution patterns within the pleural cavity, where integrating sphere effects dominate light propagation. Accurate prediction of light fluence rate distributions is essential for optimizing treatment protocols and improving therapeutic outcomes in this emerging clinical application.

**Aim:**

The aim is to quantitatively analyze light fluence rate distributions in pleural PDT using Monte Carlo (MC) simulations in various cavity geometries and tissue optical properties, providing essential data for treatment planning.

**Approach:**

Graphics processing unit-accelerated MC simulations ($10^{8}\;\text{photons}$) using MCmatlab analyzed light distribution in spherical cavities (radii 0.2 to 10 cm) and anatomically realistic lung cavity models (volume = 2 L) with point sources. Simulations include a range of tissue optical properties ($\mu_{a}$: 0.1 to $1.0\;{\mathrm{cm}}^{-1}$; $\mu_{s}^{\prime }$: 5 to $40\;{\mathrm{cm}}^{-1}$) for a flat-cut fiber source inside a realistic three-dimensional (3D) lung geometry, including realistic thoracotomy access openings and different fill media (air versus saline). Experimental validation is made using isotropic detectors in the same 3D-printed lung phantom with varying optical properties.

**Results:**

MC statistical uncertainties averaged 1.9% across all voxels. Spherical cavities (r=4  cm) demonstrated highly uniform scattered light distribution along cavity–tissue boundaries (distribution uniformity 4.9%), whereas anatomically realistic lung phantoms showed greater heterogeneity (49.9%). Scattered light fluence rate per source power ($\phi_{s}/S$) strongly correlated with tissue optical properties, particularly scattering coefficients. Source position minimally affected scattered light patterns, though direct components remained position-dependent. Side openings reduced scatter fluence near access points, with saline-filled cavities showing slightly higher fluence rates than air-filled cavities.

**Conclusions:**

We demonstrate that patient-specific factors including cavity geometry, tissue optical properties, and surgical access considerations significantly influence light distribution in pleural PDT. The quantitative relationships established between these parameters and fluence patterns provide essential data for developing personalized treatment planning protocols to optimize therapeutic light delivery.

## 1 Introduction

Photodynamic therapy (PDT) has gained significant attention as an adjuvant treatment for malignant pleural diseases, particularly when integrated with surgical interventions. The synergistic combination of surgical resection and PDT shows promise for malignant pleural mesothelioma, with clinical investigations demonstrating improvements in both local control and patient survival metrics.^1^^–^^4^ This therapeutic approach utilizes the ability of photosensitizing agents to selectively accumulate in tumor cells, which, when activated by specific wavelengths of light, generate cytotoxic reactive oxygen species that induce tumor cell death while potentially sparing normal tissues.^5^^,^^6^ The effectiveness of pleural PDT depends on precise and uniform delivery of light throughout the treatment cavity, a challenge complicated by the irregular anatomy of the pleural space and heterogeneous optical properties of surrounding tissues.^7^^–^^9^

When considering light propagation within the pleural cavity during PDT, the total fluence rate comprises two fundamental components: direct light emanating from the source and scattered light resulting from tissue interactions. Although direct light follows predictable inverse-square attenuation patterns, the scattered component demonstrates complex behavior strongly influenced by tissue optical characteristics and cavity geometry.^10^^,^^11^ This scattered light component becomes increasingly dominant at greater distances from the source and plays a critical role in delivering therapeutic light to regions not directly illuminated, which is a particularly important consideration given the practical limitations of source positioning during treatment.

Current clinical approaches to pleural PDT typically employ isotropic point sources that are systematically moved throughout the cavity, with light dosimetry performed using strategically positioned detectors.^10^^,^^12^ However, this approach provides limited spatial resolution of actual light distribution and may not adequately account for the heterogeneous nature of the treatment environment. Clinical observations suggest significant variability in treatment response, which may partially result from inconsistent light delivery across the treatment surface.^13^^,^^14^

Computational modeling, particularly Monte Carlo (MC) simulation, offers a powerful tool for investigating the complex light-tissue interactions in pleural PDT.^15^ The present study employs graphics processing unit (GPU)-accelerated MC simulations to systematically analyze light fluence rate distributions in both idealized spherical cavities and anatomically realistic lung models. We also compare MC results with analytical predictions based on integrating sphere theory to validate computational approaches and establish their applicability limits. By comparing these geometries across varying optical properties and source positions, we aim to elucidate the fundamental determinants of light distribution patterns in pleural PDT.

Through this comprehensive analysis, we seek to establish quantitative relationships that can inform more patient-specific approaches to pleural PDT planning and delivery.^16^^,^^17^ By understanding the interplay between geometric factors and optical properties, clinicians may better optimize treatment parameters to deliver uniform cumulative light fluence, potentially improving therapeutic outcomes and reducing treatment-related complications.

## 2 Materials and Methods

### 2.1 Theoretical Framework for Light Fluence Rate Distribution

The quantitative analysis of light distribution in pleural PDT requires consideration of both direct and scattered light components.^18^ The light fluence rate (ϕ, measured in $\mathrm{mW}/{\mathrm{cm}}^{2}$) directly determines the rate of photosensitizer activation and subsequent reactive oxygen species generation,^19^^,^^20^ making accurate fluence rate quantification essential for predicting therapeutic efficacy and optimizing treatment parameters. The light fluence rate to each point on the cavity is a sum of the primary component and the scattered component of the light. Primary light travels from a source to a point without scattering and is characterized by attenuation and the inverse square law. In contrast, scattered light is indirect, originating from the source but scattering off surrounding structures before reaching the point^21^^–^^24^

$$\phi_{\mathrm{tot}}=\phi_{p}+\phi_{s}.$$ (1)

The primary light component ($\phi_{p}$) follows established physical principles for photon propagation from a point source and can be modeled as

$$\phi_{p}=\frac{S}{4\pi r^{2}}A(\theta ),$$ (2)

where S represents source power, r denotes the distance from the source to the point of interest, and A(θ) accommodates the angular dependence of source emission.

The scattered light component ($\phi_{s}$) exhibits more complex behavior due to multiple reflection and scattering events within the enclosed cavity. For analytical approximation, the pleural cavity can be expressed as an integrating sphere, yielding^18^

$$\phi_{s}=\frac{4S}{A_{s}}\cdot \frac{\rho }{1-\rho (1-f)}.$$ (3)

Here, $A_{s}$ represents the total surface area of the cavity, ρ denotes the diffuse reflectance coefficient at the tissue interface, and f indicates the fraction of open surface area (set to 0 and 0.1 in our closed and side opening cavity simulations, respectively). This expression derives from the geometric series:^18^ $\phi =(4S/A_{s})\times \rho \times (1+\rho (1-f)+\rho^{2}(1-f{)}^{2}+\ldots )$, where after each reflection, fraction f escapes through the opening while (1−f) undergoes further scattering, ρ can be calculated based on diffusion theory for a semi-infinite medium.^25^ The diffuse reflectance parameter encapsulates the optical properties of surrounding tissues and serves as a critical determinant of scattered light behavior.

### 2.2 Simulation Geometries

**Fig. 1:**
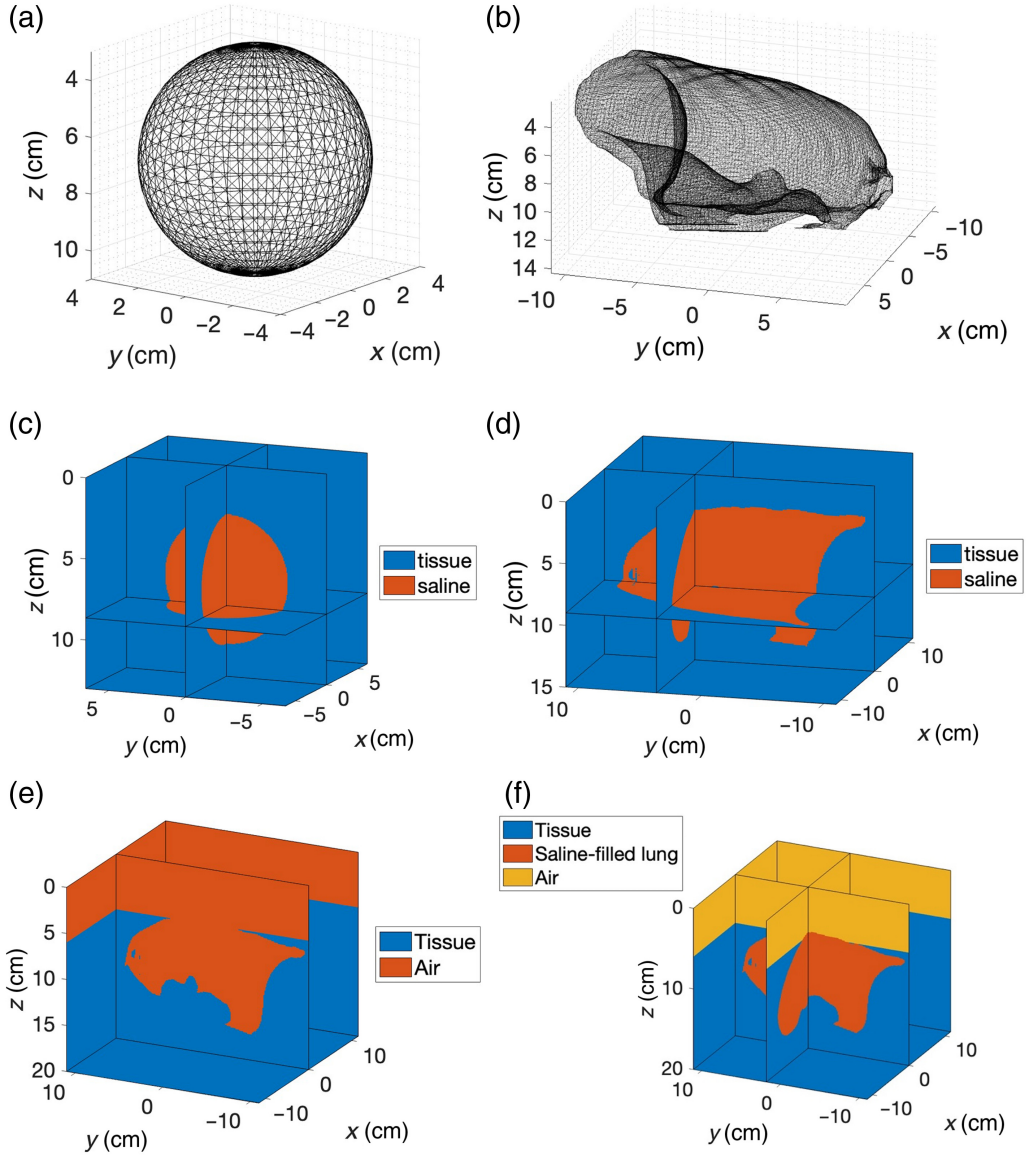
Simulation geometries for light fluence rate distribution analysis in pleural PDT. (a) Sphere cavity mesh: mesh representation of the spherical cavity model with a 4-cm radius used for theoretical validation. (b) Lung cavity mesh: mesh representation of the anatomically realistic lung cavity derived from patient data, showing the complex surface topology of the pleural space. (c) Sphere geometry: the spherical model implemented in the Monte Carlo simulation, showing the cavity (orange) surrounded by tissue (blue). (d) Lung geometry: the basic lung phantom model showing the saline-filled cavity (orange) surrounded by tissue (blue). (e) Lung (air) geometry: enhanced lung model incorporating a side opening with air (orange) surrounded by tissue (blue) to simulate the thoracotomy incision site. (f) Lung (air–saline) geometry: clinical treatment model with saline-filled lung cavity (orange) and surrounding air layer (yellow) at the thoracotomy site, all encompassed by tissue (blue).

To investigate light distribution patterns under controlled conditions while maintaining clinical relevance, we developed multiple geometric models (Fig. 1). Initially, we studied two basic configurations: a spherical cavity [Figs. 1(a) and 1(c)] and an anatomically realistic lung cavity [Figs. 1(b) and 1(d)], both surrounded by homogeneous tissue media (refractive index 1.4) and filled with saline (refractive index 1.33).^26^^,^^27^ The properties of both geometries are presented in Table 1.

**Table 1:** Summary of radius, surface area, and volume for both geometries.

Geometry	Radius^a^ (cm)	Surface area (${\mathrm{cm}}^{2}$)	Volume (${\mathrm{cm}}^{3}$)
Sphere	4	201.1	268.1
Lung	7.9	780.5	1123.7

a Radius for lung refers to the equivalent radius ($=\sqrt{A/4\pi }$), where A is the surface area).

The spherical model utilized a cavity with a 4-cm radius, providing an idealized geometry for theoretical validation and systematic parameter analysis. This simplified configuration enabled direct comparison with integrating sphere theory while eliminating confounding variables associated with irregular surfaces. However, the substantial geometric complexity of actual pleural cavities, as demonstrated by our anatomically realistic models, necessitates patient-specific computational approaches rather than simplified geometric approximations for clinical applications. Our anatomically realistic model incorporated a detailed pleural cavity structure derived from patient imaging data. This phantom replicated the complex surface topology characteristic of the pleural space.

To better approximate clinical conditions, we subsequently developed more sophisticated models with a side opening to simulate the thoracotomy incision site present during actual treatments. The first variation [Fig. 1(e)] extended the basic lung cavity model to include an air region representing the open thoracic cavity exposed during surgery. The second variation [Fig. 1(f)] represented the fluid-filled state used during clinical PDT treatments, with saline filling the lung cavity while maintaining the air interface at the thoracotomy site. These models incorporated realistic boundary conditions at different material interfaces (tissue–air, tissue–saline, and air–saline), enabling comprehensive investigations of how the presence of an opening and different filling media affect scattered light distribution and overall treatment uniformity.

Geometric configurations were implemented within computational volumes comprising 200×200×200  voxels for spherical models and 250×250×200  voxels for the anatomically realistic pleural cavity models, with the voxel size of 0.06 cm. This discretization provided sufficient spatial detail to capture relevant anatomical features while maintaining computational efficiency.

### 2.3 Monte Carlo Implementation

Our light propagation simulations employed a GPU-accelerated MC approach (MCmatlab) to model photon transport through heterogeneous media.^28^ The simulation platform was developed based on established photon migration algorithms with modifications to optimize performance for the specific geometries and boundary conditions relevant to pleural PDT.^29^ GPU acceleration enabled the tracking of $10^{8}\;\text{photons}$ per simulation, providing statistically robust results while maintaining reasonable computation times (average 45 min per simulation on an NVIDIA GeForce RTX 2080 GPU).

In this study, we implemented a flat-cut fiber with emission constrained to a 30-deg solid angle, with the numeric aperture (NA) = 0.5.^30^ The irradiation wavelength was 632 nm. The irradiation wavelength was 632 nm, consistent with clinical applications. Our forward-directed beam approach was chosen as a practical solution to achieve reliable component separation within the constraints of the available framework.^31^

Although this differs from the isotropic source used in experimental validation, this approach enables more precise analysis of light distribution mechanisms while maintaining clinical relevance, as the fundamental physics governing scattered light behavior remains applicable to clinical scenarios. Our experimental two-step measurement protocol (described in Sec. 2.4) directly validates this computational approach by isolating scattered light components through subtraction methods.^32^

We conducted a parametric investigation across a range of tissue optical properties, with absorption coefficients ($\mu_{a}$) varying from 0.1 to $1.0\;{\mathrm{cm}}^{-1}$ in $0.1\text{-}{\mathrm{cm}}^{-1}$ increments and reduced scattering coefficients ($\mu_{s}^{\prime }$) ranging from 5 to $40\;{\mathrm{cm}}^{-1}$ in $5\text{-}{\mathrm{cm}}^{-1}$ increments.^33^^,^^34^ These ranges encompass the values typically observed in pleural tissues and surrounding thoracic structures. For the closed cavity model, simulations were performed with three distinct source positions: central (0,0,0), Y-shifted (−5  cm), and Z-shifted (+2  cm). This positioning strategy allowed assessment of location-dependent effects on light distribution patterns.

The simulation algorithm incorporated key physical phenomena governing light propagation in biological tissues, including photon scattering based on the Henyey–Greenstein phase function with anisotropy factor g=0.9, boundary interactions with appropriate Fresnel reflection and transmission at media interfaces, absorption-dependent photon weight adjustment using established variance reduction techniques, and position-dependent fluence rate scoring throughout the computational domain.^35^ The spatial heterogeneity (coefficient of variation) was evaluated by calculating the coefficient of variation across multiple independent simulation runs, with particular attention to regions relevant for clinical dosimetry.

The statistical uncertainty of Monte Carlo simulations was evaluated using established methods for photon-based simulations. For each voxel, the relative statistical uncertainty was calculated as 1/√n, where n represents the number of photons contributing to that voxel. This approach provides an estimate of simulation convergence and distinguishes statistical uncertainty from spatial variability measures used to quantify light distribution heterogeneity across boundary positions.^36^

### 2.4 Phantom Measurements

**Fig. 2:**
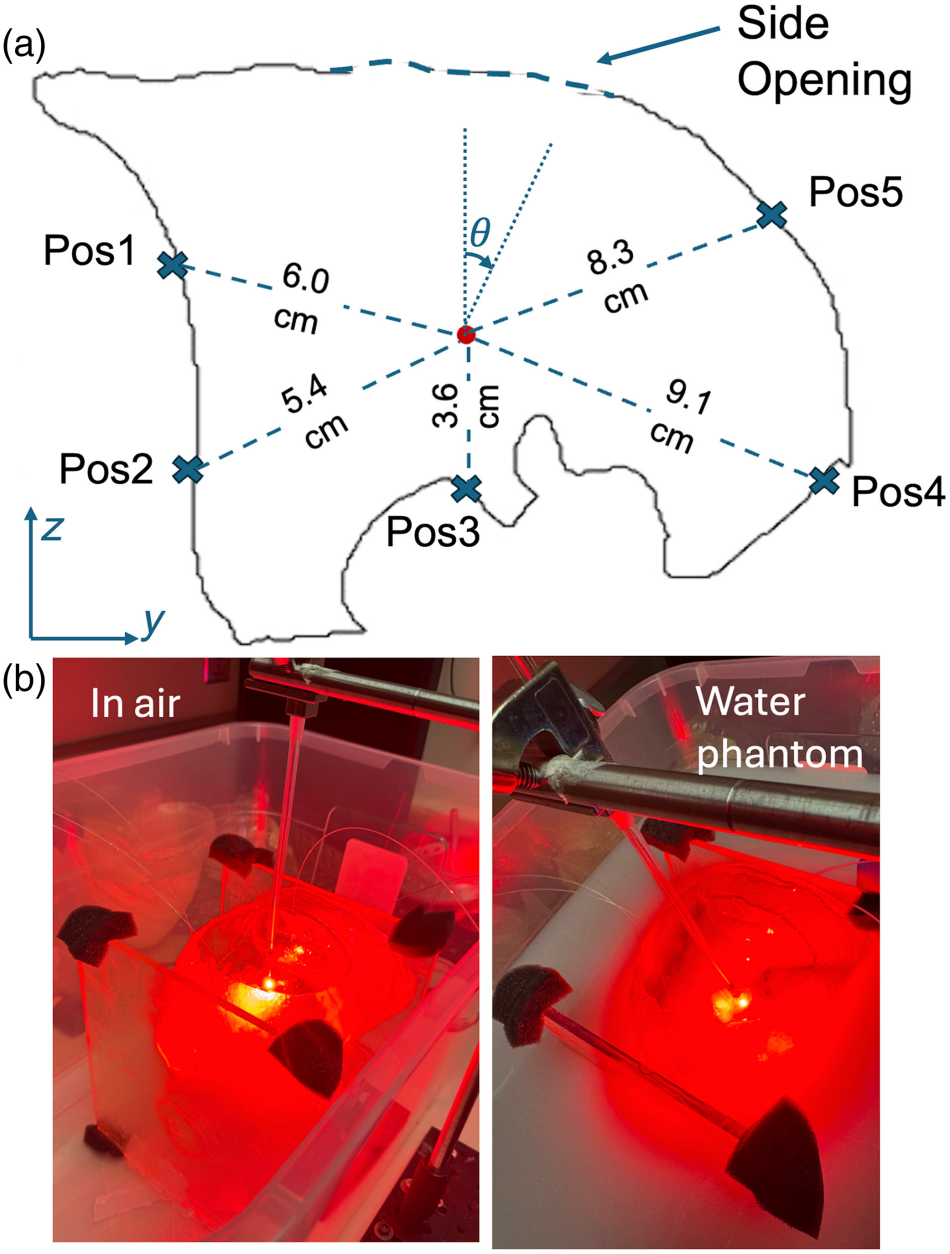
(a) Schematics for the center light source positions for the 2-L lung phantom (1 L per lung) and the locations of the isotropic detectors. (b) Experimental setup of light fluence rate measurement.

To validate our simulation results, we conducted experimental measurements using a three-dimensional (3D)-printed anatomical lung phantom developed specifically for our pleural PDT study.^37^ The 3D-printed photopolymerized resin phantom was designed to be thin and transparent to minimize optical interference, though residual effects from the phantom material may contribute minor measurement errors. Our group has previously demonstrated the validity of theoretical approaches using an ellipsoid phantom.^31^ The phantom was fabricated based on the same geometry used in our computational model [Fig. 1(c)], with a 2-L internal volume (1 L per lung) and a side opening to replicate the thoracotomy access site. We performed measurements across a range of tissue optical properties by systematically varying the concentrations of Black India ink (Higgins Inc., Leeds, Massachusetts, United States) and Nutralipid (20%, B. Braun Medical Inc., Bethlehem, Pennsylvania, United States) in a liquid medium surrounding the lung phantom to adjust $\mu_{a}$ and $\mu_{s}^{\prime }$.^38^ Fluence rate measurements were obtained at five pre-determined locations distributed throughout the cavity [Fig. 2(a)], with positions selected to capture both near-field and far-field regions relative to the source. These five detector positions were selected to validate computational models under controlled conditions rather than to replicate clinical measurement protocols. In the clinical pleural PDT clinical trial, light fluence is measured at eight standardized anatomical sites to ensure adequate dose delivery to critical pleural surfaces.^8^ The phantom detector locations were chosen based on experimental accessibility with the rigid phantom and to represent geometrically distinct regions, including areas near the thoracotomy opening, smooth surfaces, and irregular contours. These measurement locations correspond to specific angular positions (θ, starting from the top as 0 deg and clockwise) around the cavity: position 1 (285 deg), position 2 (240 deg), position 3 (180 deg), position 4 (120 deg), and position 5 (55 deg).

The experimental setup [Fig. 2(b)] consisted of an isotropic point source positioned at the center of the lung phantom cavity. The experimental setup accounted for this source configuration difference through a systematic two-step measurement protocol designed to isolate scattered light components. First, in-air measurements at all detector positions quantified the direct light component from the isotropic source. Although Fig. 2(b) shows the experimental setup, any environmental scattering from phantom materials, supports, or containers was minimal and consistent across all measurements, allowing for reliable subtraction analysis to isolate tissue-induced scattered light components. Subsequently, measurements with tissue-simulating phantom medium captured the total fluence rate, enabling scattered light calculation by subtraction.

For each optical property combination, we first conducted in-air measurements at all five detector positions to quantify the direct light component. Subsequently, we performed measurements with the cavity surrounded by the prepared optical phantom medium to capture the total fluence rate (direct + scattered components). The scattered light component was then calculated by subtracting the direct light contribution from the total measured fluence rate. This experimental validation approach allowed direct comparison between simulated and measured fluence rate distributions, providing a critical assessment of our computational model’s accuracy under conditions approximating clinical scenarios.

### 2.5 Analytical Methods

To comprehensively characterize light distribution, we employed several analytical methods. Boundary fluence analysis quantified fluence rate profiles along the cavity boundary relative to the light source, isolating scattered light from direct illumination peaks and visualizing the distribution to identify potential under-dosed regions for treatment planning. For assessing treatment uniformity, we analyzed the spatial distribution of scattered light fluence rate across the cavity boundary. This involved excluding regions with over 10% direct light contribution and calculating the coefficient of variation (standard deviation/mean) for the remaining boundary, where lower values indicate greater uniformity. This analysis was performed for each combination of optical properties and source positions, and statistical comparisons were made between spherical and anatomically realistic models to quantify the impact of geometric complexity on uniformity.

The relationship between cavity size and light distribution was investigated by systematically varying the radius of the spherical cavity (0.5 to 10 cm) and applying proportional scaling to the anatomically realistic model. For multiple combinations of absorption ($\mu_{a}=0.3$, $0.7\;{\mathrm{cm}}^{-1}$) and reduced scattering ($\mu_{s}^{\prime }=100$, $300\;{\mathrm{cm}}^{-1}$) coefficients, and three source positions (central, y-shifted, and z-shifted), we calculated the normalized fluence rate and compared results to the integrating sphere model. Finally, to validate the computational model, we extracted simulated total and scattered fluence rate values at five specific points corresponding to experimental detector positions within the lung phantom. This allowed for direct point-by-point comparison with experimental measurements to assess the model’s predictive accuracy.

## 3 Results

### 3.1 Validation of Simulation with Experimental Measurements and Theoretical Calculations

Our initial validation of the Monte Carlo simulation approach involved comparing the computational results with theoretical predictions for an idealized spherical cavity. Table 2 presents a comparison between experimental measurements and simulation results for three representative optical property combinations. The average values of those measured at these five discrete positions will be compared with simulation results and theoretical predictions in the results section to provide comprehensive validation of our computational model.

**Table 2:** Comparison of scatter light fluence rates per source power, $\phi_{s}/S$ (${\mathrm{cm}}^{-2}$) at five detector positions between measurement and simulation for three representative optical property combinations in the anatomically realistic lung phantom with side opening: (a) measurements, (b) MC simulations, (c) % diff (MC/Meas-1). Uncertainties for measurements represent standard deviations across three repeated measurements at each detector position. Uncertainties for simulations represent statistical uncertainties based on photon count statistics (1/√n method) for the corresponding spatial locations.

Position	$\mu_{a}=0.1$, $\mu_{s}^{\prime }=20$	$\mu_{a}=0.3$, $\mu_{s}^{\prime }=30$	$\mu_{a}=0.7$, $\mu_{s}^{\prime }=40$	In-air
Selected measurement data				
Pos1	0.014 ± 0.002	0.0076 ± 0.0008	0.0058 ± 0.0006	0.0023 ± 0.0002
Pos2	0.013 ± 0.002	0.0081 ± 0.0009	0.0058 ± 0.0007	0.0026 ± 0.0003
Pos3	0.013 ± 0.001	0.0085 ± 0.0008	0.0057 ± 0.0005	0.0062 ± 0.0005
Pos4	0.0094 ± 0.0008	0.0068 ± 0.0006	0.0044 ± 0.0004	0.0009 ± 0.0001
Pos5	0.0096 ± 0.0009	0.0067 ± 0.0007	0.0042 ± 0.0005	0.0013 ± 0.0002
Selected simulation data				
Pos1	0.015 ± 0.0008	0.0088 ± 0.0005	0.0057 ± 0.0003	
Pos2	0.015 ± 0.0007	0.0086 ± 0.0004	0.0049 ± 0.0003	
Pos3	0.014 ± 0.0006	0.0089 ± 0.0005	0.0061 ± 0.0004	
Pos4	0.010 ± 0.0005	0.0054 ± 0.0003	0.0036 ± 0.0002	
Pos5	0.0097 ± 0.0005	0.0058 ± 0.0003	0.0037 ± 0.0002	
Percentage difference				
Pos1	7.1%	15.8%	−1.7%	
Pos2	15.4%	6.2%	−15.5	
Pos3	7.7%	4.7%	7.0%	
Pos4	6.4%	−20.6%	−18.2%	
Pos5	1.0%	−13.4%	−11.9%	

Table 3 presents this comparison across a range of optical properties, with Monte Carlo simulations (Table 3a) showing excellent agreement with theoretical predictions based on the integrating sphere model (Table 3b). Table 4 presents the comparison of scattered light fluence rates across multiple optical property combinations, showing measured values (Table 4a), simulation results (Table 4b), and theoretical predictions based on Eq. (3) (Table 4c) with f=0.1 to account for the side opening. The values presented in Tables 4a and 4b represent the mean scattered fluence rates averaged across all five measurement locations within the lung phantom for each optical property combination, with standard deviations indicating the inter-position variability. Table 5 further quantifies these relationships through percentage differences among different methods.

**Table 3:** Comparison of boundary fluence rates per source power, ϕ/S, (${\mathrm{cm}}^{-2}$) for a spherical cavity. (a) Monte Carlo simulation results showing mean ± standard deviation across the cavity boundary for different optical property combinations. Statistical uncertainties from Monte Carlo simulations averaged 1.93%. (b) Theoretical predictions using the modified integrating sphere model [Eq. (3)] with f=0 (no opening), where the diffuse reflectance ρ depends on both $\mu_{a}$ and $\mu_{s}^{\prime }$ according to $\rho =\mu_{s}^{\prime }/(\mu_{a}+\mu_{s}^{\prime })$.

$\mu_{a}/\mu_{s}^{\prime }$ (${\mathrm{cm}}^{-1}$)	5	10	15	20	30	40
(a) MC simulations						
0.1	0.022 ± 0.005	0.033 ± 0.0035	0.041 ± 0.003	0.049 ± 0.003	0.060 ± 0.003	0.069 ± 0.004
0.3	0.011 ± 0.003	0.018 ± 0.002	0.022 ± 0.001	0.026 ± 0.001	0.032 ± 0.002	0.036 ± 0.002
0.5	0.0089 ± 0.0020	0.013 ± 0.001	0.016 ± 0.001	0.019 ± 0.001	0.023 ± 0.002	0.027 ± 0.002
0.7	0.0071 ± 0.0015	0.011 ± 0.001	0.013 ± 0.001	0.015 ± 0.001	0.019 ± 0.001	0.022 ± 0.002
1.0	0.0058 ± 0.0055	0.0090 ± 0.0008	0.011 ± 0.001	0.012 ± 0.001	0.015 ± 0.001	0.018 ± 0.002
(b) Theory [Eq. (3), f=0]						
0.1	0.022	0.032	0.040	0.046	0.058	0.067
0.3	0.011	0.017	0.022	0.025	0.032	0.037
0.5	0.0084	0.013	0.016	0.019	0.024	0.028
0.7	0.0069	0.010	0.013	0.016	0.019	0.023
1.0	0.0055	0.0084	0.011	0.013	0.016	0.019

**Table 4:** Comparison of scattered light fluence rates per source power, $\phi_{s}/S$, (${\mathrm{cm}}^{-2}$) for the lung phantom with a side opening across various optical properties: (a) experimental measurements from the 3D-printed phantom (mean from measurements at five discrete positions), (b) Monte Carlo simulation results (mean from values at five discrete positions), and (c) theoretical predictions using the modified integrating sphere model [Eq. (3)] with f=0.11. Values for measurements and simulations represent the mean ± standard deviation across five detector positions within the cavity.

$\mu_{a}/\mu_{s}^{\prime }$ (${\mathrm{cm}}^{-1}$)	5	10	15	20	30	40
(a) Measurements						
0.1	0.0083 ± 0.0014	0.012 ± 0.002	0.014 ± 0.002	0.017 ± 0.002	0.019 ± 0.003	0.022 ± 0.004
0.3	0.0038 ± 0.0011	0.0060 ± 0.0008	0.0075 ± 0.0008	0.0097 ± 0.001	0.012 ± 0.001	0.0141 ± 0.001
0.5	0.0024 ± 0.0009	0.0040 ± 0.0009	0.0050 ± 0.0011	0.0063 ± 0.0009	0.0075 ± 0.0008	0.0089 ± 0.0010
0.7	0.0021 ± 0.0008	0.0032 ± 0.0006	0.0041 ± 0.0007	0.0050 ± 0.0007	0.0060 ± 0.0006	0.0066 ± 0.0009
1.0	0.0013 ± 0.0005	0.0021 ± 0.0004	0.0031 ± 0.0005	0.0036 ± 0.0007	0.0041 ± 0.0009	0.0047 ± 0.0010
(b) MC simulations						
0.1	0.0083 ± 0.0018	0.012 ± 0.001	0.015 ± 0.002	0.017 ± 0.0026	0.020 ± 0.003	0.022 ± 0.004
0.3	0.0037 ± 0.0012	0.0060 ± 0.0014	0.0076 ± 0.0017	0.0090 ± 0.0021	0.011 ± 0.002	0.012 ± 0.003
0.5	0.0024 ± 0.0010	0.0040 ± 0.0011	0.0053 ± 0.0013	0.0064 ± 0.0017	0.0078 ± 0.0018	0.0088 ± 0.0023
0.7	0.0018 ± 0.0008	0.0031 ± 0.0010	0.0041 ± 0.0011	0.0050 ± 0.0013	0.0062 ± 0.0014	0.0071 ± 0.0020
1.0	0.0013 ± 0.0005	0.0023 ± 0.0007	0.0031 ± 0.0007	0.0036 ± 0.0008	0.0046 ± 0.0009	0.0054 ± 0.0011
(c) Theory [Eq. (3), f=0.1]						
0.1	0.0071	0.011	0.013	0.015	0.018	0.020
0.3	0.0033	0.0055	0.0071	0.0084	0.011	0.012
0.5	0.0022	0.0039	0.0051	0.0062	0.0079	0.0093
0.7	0.0016	0.0030	0.0041	0.0050	0.0065	0.0077
1.0	0.0010	0.0022	0.0031	0.0039	0.0051	0.0062

**Table 5:** Percentage differences among methods for scattered light fluence rates per source power, $\phi_{s}/S$, (${\mathrm{cm}}^{-2}$) across various optical properties and geometries.

$\mu_{a}/\mu_{s}^{\prime }$ (${\mathrm{cm}}^{-1}$)	5	10	15	20	30	40
(a) Theory [Eq. (3), f=0] versus MC for spherical cavity (%)						
0.1	0.5%	−3.0%	−2.9%	−5.9%	−3.8%	−3.5%
0.3	−2.7%	−5.0%	−1.3%	−3.1%	1.6%	3.1%
0.5	−5.7%	−3.0%	−2.4%	0.5%	4.8%	5.7%
0.7	−3.1%	−5.7%	−3.0%	3.9%	2.7%	5.0%
1.0	−5.3%	−6.9%	0.9%	4.8%	4.6%	5.0%
(b) MC versus measurement for lung phantom (%)						
0.1	0.1%	6.1%	6.3%	3.0%	5.8%	0.9%
0.3	−2.9%	−1.3%	2.4%	−7.2%	−7.8%	−14.2%
0.5	1.7%	1.8%	6.0%	1.3%	3.9%	−0.8%
0.7	−12.1%	−1.6%	0.7%	−0.8%	3.0%	7.4%
1.0	−4.5%	8.5%	−1.0%	0.6%	14.1%	15.4%
(c) Theory [Eq. (3), f=0.1] versus measurement for lung phantom (%)						
0.1	−14.0%	−4.3%	−9.7%	−10.2%	−5.3%	−9.9%
0.3	−12.9%	−8.8%	−4.8%	−13.1%	−5.2%	−14.9%
0.5	−8.0%	−1.3%	2.2%	−1.4%	5.8%	4.4%
0.7	−22.7%	−5.0%	−0.2%	−0.2%	7.8%	16.8%
1.0	−24.8%	3.3%	−0.3%	8.6%	25.9%	32.2%
(d) Theory [Eq. (3), f=0.1] versus MC for lung phantom (%)						
0.1	−14.0%	−9.8%	−15.0%	−12.8%	−10.4%	−10.7%
0.3	−10.3%	−7.6%	−7.1%	−6.4%	2.8%	−0.8%
0.5	−9.5%	−3.0%	−3.6%	−2.7%	1.8%	5.2%
0.7	−12.1%	−3.5%	−0.9%	0.6%	4.7%	8.8%
1.0	−21.3%	−4.8%	0.7%	8.0%	10.4%	14.6%

### 3.2 Boundary Fluence Rate Distribution in Different Cavity Models

**Fig. 3:**
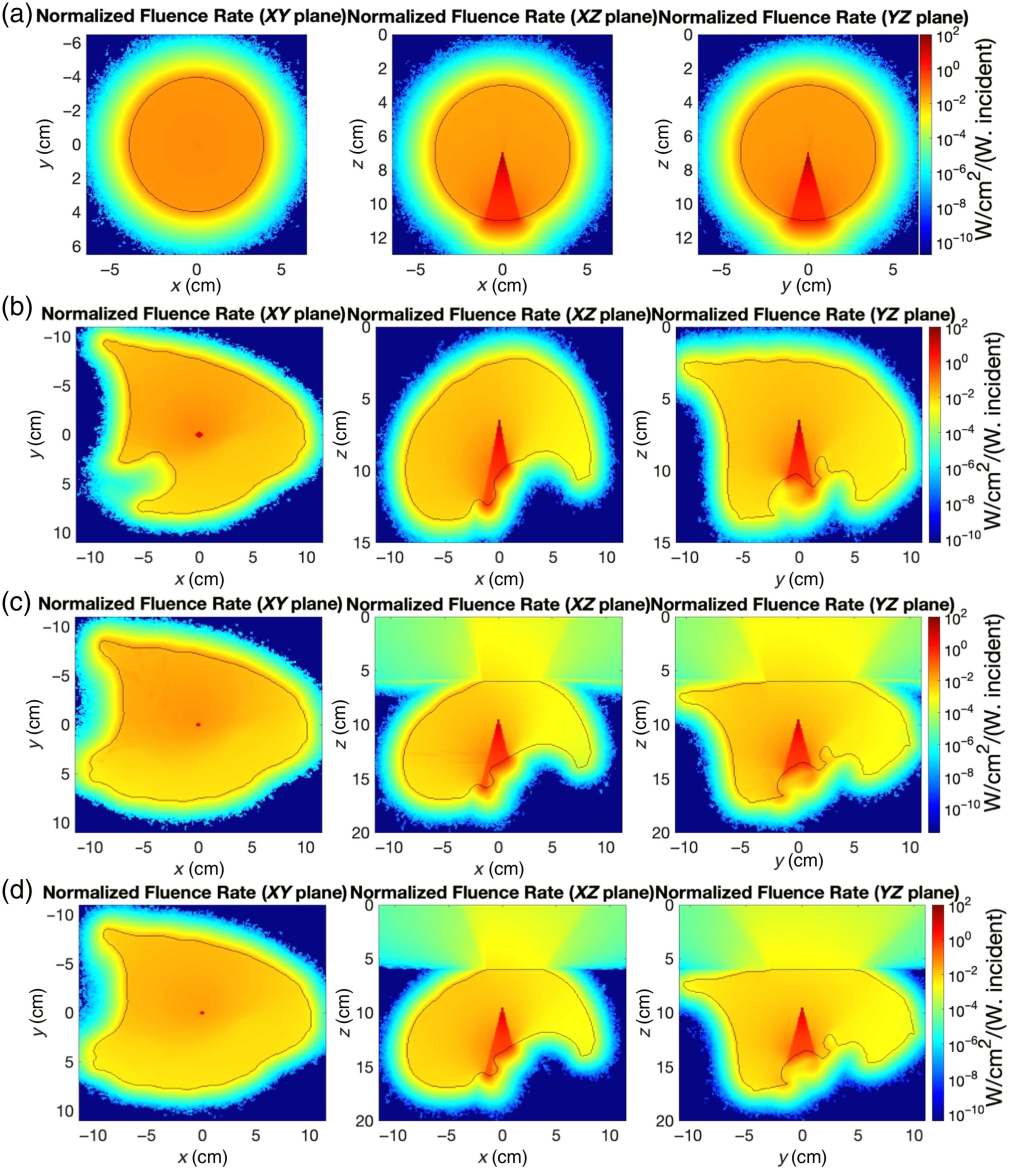
Normalized fluence rate distributions in different simulation geometries for tissue with $\mu_{a}=0.5\;{\mathrm{cm}}^{-1}$ and $\mu_{s}^{\prime }=15\;{\mathrm{cm}}^{-1}$. (a) Spherical cavity model (r=4  cm). (b) Anatomically realistic lung cavity phantom. (c) Lung cavity phantom with air opening (volume = 1 L). (d) Saline-filled lung cavity phantom with an air opening. Each row displays cross-sectional views in three orthogonal planes (XY, XZ, and YZ). Dashed/solid lines indicate the cavity–tissue boundary.

Figure 3 illustrates the normalized fluence rate distributions across our different simulation geometries for representative optical properties ($\mu_{a}=0.5\;{\mathrm{cm}}^{-1}$; $\mu_{s}^{\prime }=15\;{\mathrm{cm}}^{-1}$). The cross-sectional views in three orthogonal planes reveal the spatial heterogeneity introduced by geometric complexity. The spherical model [Fig. 3(a)] demonstrates highly symmetrical light distribution with relatively uniform fluence along the cavity boundary. In contrast, the anatomically realistic lung model [Fig. 3(b)] exhibits more complex patterns with regions of both higher and lower fluence determined by the irregular cavity geometry. When incorporating a side opening [Figs. 3(c) and 3(d)], we observe distinct alterations in the fluence pattern, particularly near the opening where scattered light escapes. The saline-filled model [Fig. 3(d)] shows subtle but clinically important differences from the air-filled version [Fig. 3(c)]. In all cases, the direct light component is visible as a conical beam emitting from the centrally positioned source, whereas the scattered light creates a more diffuse distribution that is highly dependent on the cavity geometry and surrounding tissue optical properties.

**Fig. 4:**
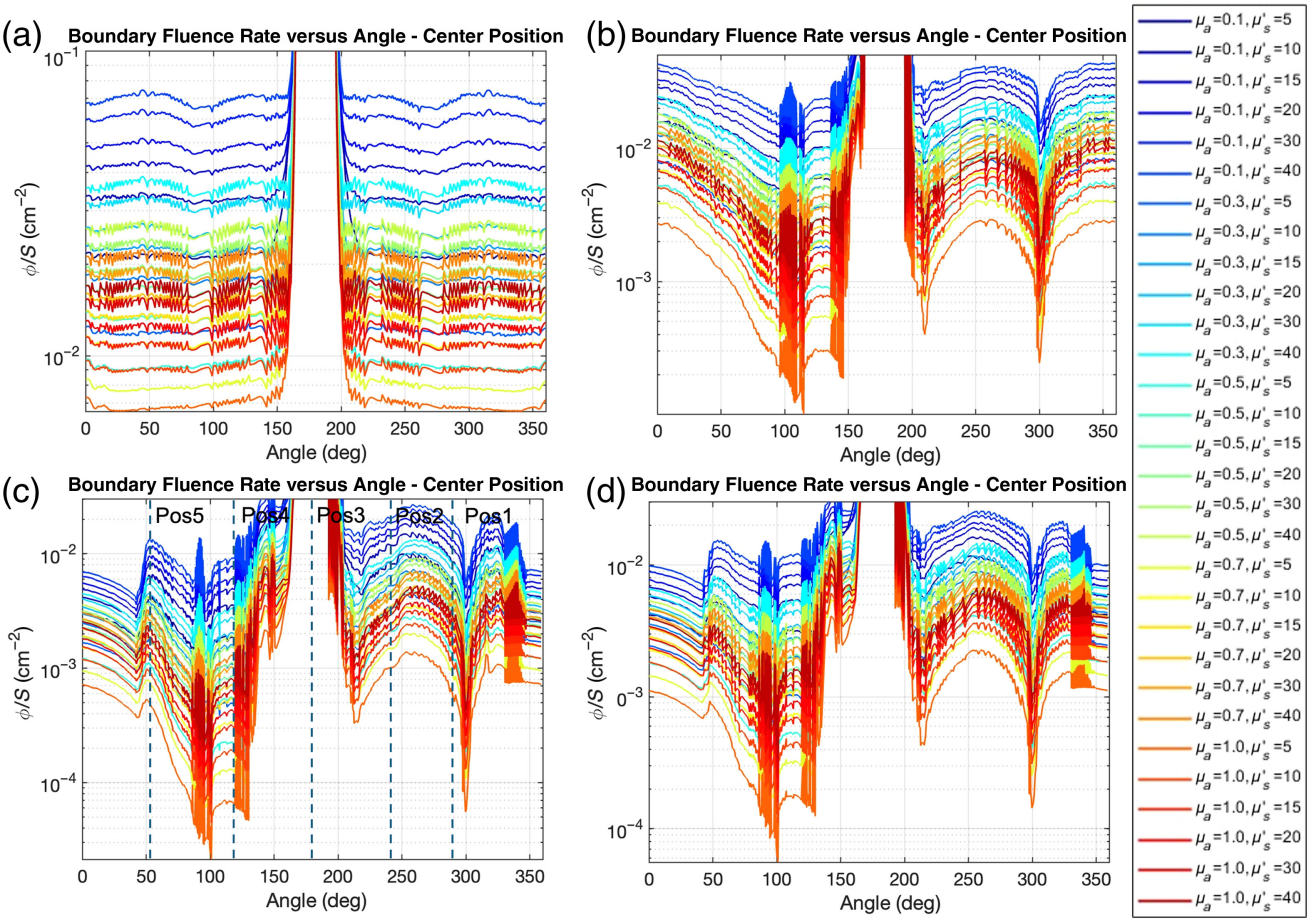
Boundary fluence rate distributions from Monte Carlo simulations as a function of angle for (a) spherical cavity model (r=4  cm), (b) anatomically realistic lung phantom, (c) lung phantom with air opening, and (d) saline-filled lung phantom with air opening. Results are shown for different combinations of optical properties with varying absorption coefficients ($\mu_{a}$: 0.1 to $1.0\;{\mathrm{cm}}^{-1}$) and reduced scattering coefficients ($\mu_{s}^{\prime }$: 5 to 40) ${\mathrm{cm}}^{-1}$) with a centrally positioned light source. The color scale progresses from dark blue (low absorption, high scattering) to red (high absorption, low scattering). The reduced fluence at 0 to 40 deg and 330 to 360 deg in panels (c) and (d), respectively, corresponds to the location of the cavity opening. The detector positions (Pos1, Pos2, Pos3, Pos4, and Pos5) in Fig. 2(a) are marked in panel (c). The disturbance of boundary fluence rate between detector Pos1 and Pos2 is likely due to an artifact in the lung phantom contour data near 95 deg [see Fig. 3(c), right figure].

Figure 4 presents boundary fluence rate distributions as a function of angle for four different simulation geometries: spherical cavity [Fig. 4(a)], anatomically realistic lung cavity [Fig. 4(b)], lung cavity with air opening [Fig. 4(c)], and lung cavity with saline filling and air opening [Fig. 4(d)]. For all four different geometries, the sharp peaks observed in angular fluence distributions represent the expected direct light signature from our forward-directed source configuration. The background levels represent scattered light distribution, which demonstrates the uniform behavior that is clinically relevant for treatment planning. Each plot shows results for various optical property combinations with absorption coefficients ranging from 0.1 to $1.0\;{\mathrm{cm}}^{-1}$ and reduced scattering coefficients from 5 to $40\;{\mathrm{cm}}^{-1}$.

In the spherical model [Fig. 4(a)], sharp peaks at 180 deg represent direct light components, with relatively uniform background levels indicating scattered light contribution. The scattered light component remains consistent across angular positions, demonstrating the symmetrical light distribution characteristic of spherical geometries. The anatomically realistic lung model [Fig. 4(b)] exhibits more complex angular dependence, with pronounced variation in both direct and scattered light components. While maintaining the central peak at 180 deg, the lung model shows additional features at ∼120 and 300 deg due to the cavity’s irregular geometry. For the lung cavity with an air opening [Fig. 4(c)], a significant reduction in scattered light fluence rate is observed in the 0- to 40-deg and 330- to 360-deg regions, corresponding to the cavity opening where boundary reflections are diminished. This pattern illustrates how the presence of an opening fundamentally alters the light distribution compared with closed cavities. The saline-filled lung model with an air opening [Fig. 4(d)] shows similar patterns to Fig. 4(c) but with slightly higher fluence rates overall.

### 3.3 Effects of Optical Properties on Boundary Fluence Rate

**Fig. 5:**
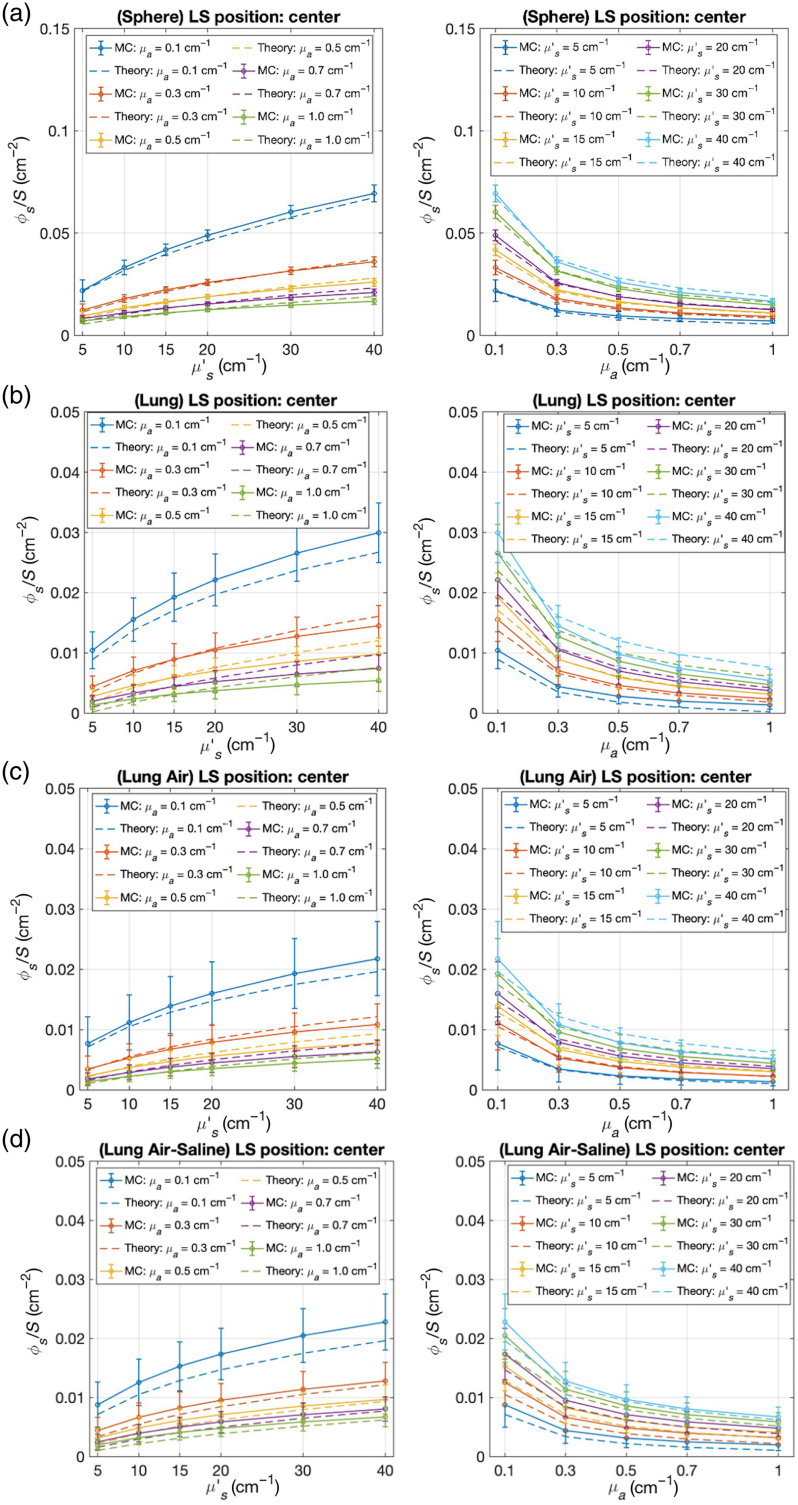
Relationship between optical properties and average ϕs/S for (a) spherical cavity (r=4  cm), (b) anatomically realistic lung cavity, (c) lung cavity with air opening, and (d) saline-filled lung cavity with air opening. Left panels show fluence rate versus reduced scattering coefficient ($\mu_{s}^{\prime }$) for different absorption coefficients; right panels show fluence rate versus absorption coefficient ($\mu_{a}$) for different scattering coefficients. Solid lines represent Monte Carlo simulation results with error bars indicating spatial variability; dashed lines show theoretical predictions based on the modified integrating sphere model, which was originally developed for spherical geometries. All results are shown for the centrally positioned light source.

Figure 5 illustrates the relationship between optical properties and boundary fluence rates for the four geometric models: spherical cavity [Fig. 5(a)], anatomically realistic lung cavity [Fig. 5(b)], lung cavity with air opening [Fig. 5(c)], and saline-filled lung cavity with air opening [Fig. 5(d)]. Each panel presents the average boundary fluence as a function of both reduced scattering coefficient ($\mu_{s}^{\prime }$, left plots) and absorption coefficient ($\mu_{a}$, right plots), with simulation results (solid lines with error bars) compared against theoretical predictions (dashed lines).

The angular distribution profiles maintain similar shapes across different optical property combinations within each geometry, but the coefficient of variation (standard deviation/mean) of scattered light fluence rate varies substantially with tissue properties (Table 6). Higher absorption coefficients consistently increase distribution heterogeneity, with anatomically realistic cavities showing coefficients of variation ranging from ∼26% to 77% depending on optical properties, whereas spherical geometries maintain better uniformity with coefficients of variation from 2% to 10%.

**Table 6:** Coefficient of variation (CV) of scattered light fluence rate across boundary positions for selected optical property combinations.

Geometry	$\mu_{a}$ (${\mathrm{cm}}^{-1}$)	$\mu_{s}^{\prime }$ (${\mathrm{cm}}^{-1}$)	CV (%)^a^
Sphere	0.1	5	3.8
	0.1	40	4.0
	0.5	5	6.2
	0.5	40	5.6
	1.0	5	10.4
	1.0	40	7.1
Lung	0.1	5	39.1
	0.1	40	26.0
	0.5	5	60.8
	0.5	40	42.1
	1.0	5	70.8
	1.0	40	52.2

a Mean CV across all combinations: sphere = 4.89% and lung = 49.9%.

### 3.4 Source Position and Size-Dependent Effect

**Fig. 6:**
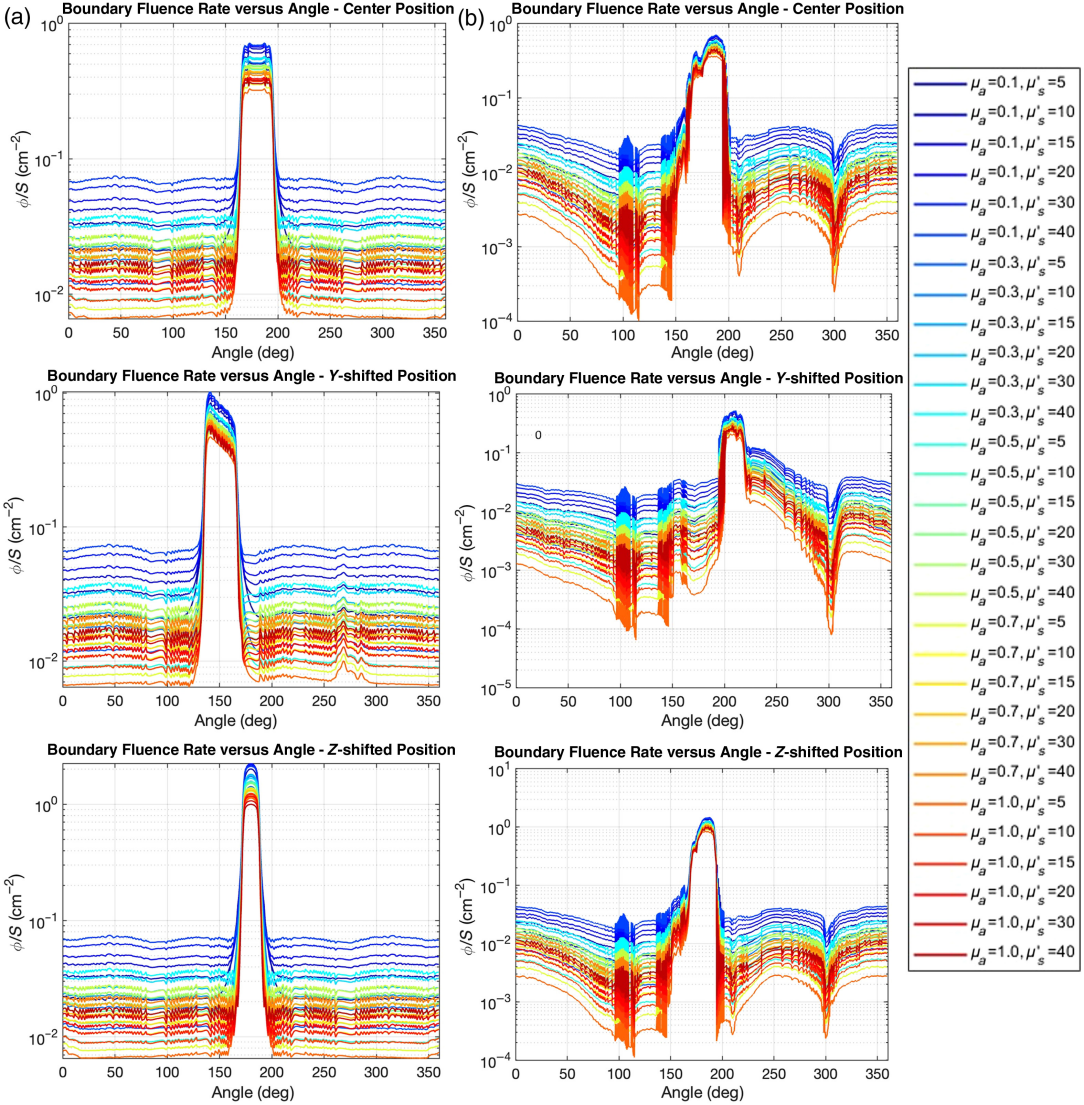
Boundary scatter fluence rate per source power distributions as a function of angle for three different source positions (center, y-shifted, and z-shifted) in (a) spherical cavity and (b) lung phantom.

Figure 6 illustrates the impact of source position on boundary fluence rate distribution for both spherical [Fig. 6(a)] and anatomically realistic lung [Fig. 6(b)] models across various optical properties. For each geometry, results are shown for three source positions: center, y-shifted (−5  cm), and z-shifted (+2  cm). In both geometries, the direct light component (represented by pronounced peaks) shifts according to source position, whereas the scattered light component (baseline levels) remains relatively uniform regardless of source placement. Despite the marked differences in peak positions and intensities, the background scattered light levels remain consistent across different source positions for each optical property combination. This pattern suggests that although direct illumination is strongly position-dependent, the scattered light contribution that dominates far-field fluence is minimally affected by source positioning.

**Fig. 7:**
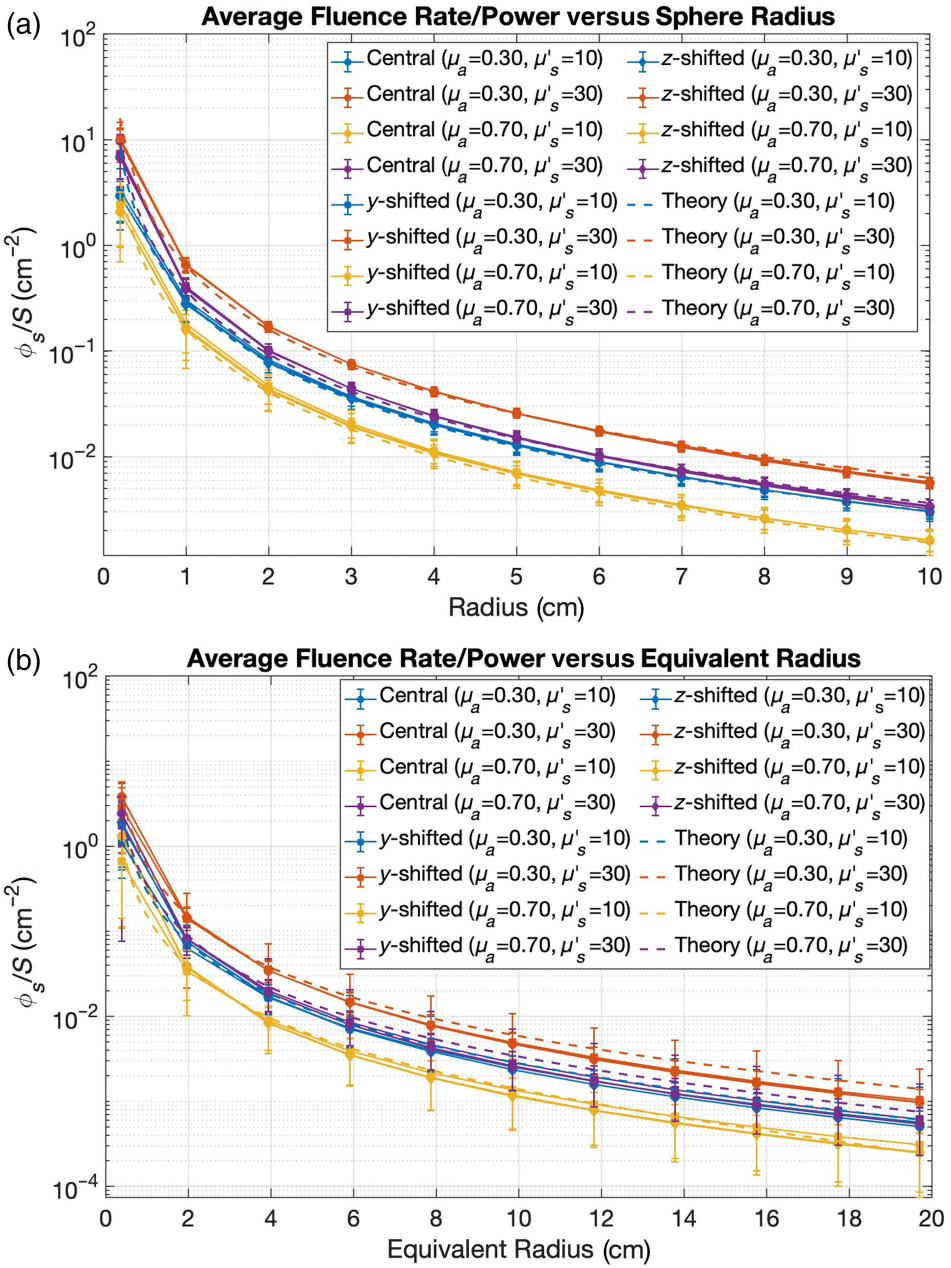
Size-dependent effects on average boundary scatter fluence rate per source power for (a) spherical cavity radius and (b) lung cavity equivalent radius. Results shown for different source positions (central, y-shifted, and z-shifted) and optical property combinations ($\mu_{a}=0.3\;{\mathrm{cm}}^{-1}$, $\mu_{s}^{\prime }=10\;{\mathrm{cm}}^{-1}$; $\mu_{a}=0.3\;{\mathrm{cm}}^{-1}$, $\mu_{s}^{\prime }=30\;{\mathrm{cm}}^{-1}$; $\mu_{a}=0.7\;{\mathrm{cm}}^{-1}$, $\mu_{s}^{\prime }=10\;{\mathrm{cm}}^{-1}$; and $\mu_{a}=0.7\;{\mathrm{cm}}^{-1}$, $\mu_{s}^{\prime }=30\;{\mathrm{cm}}^{-1}$). Dashed lines represent theoretical predictions. Error bars indicate spatial variability of fluence rates across the cavity boundary.

Figure 7 presents the relationship between cavity size and average boundary fluence rate for spherical [Fig. 7(a)] and lung cavity models [Fig. 7(b)]. The equivalent radius of the anatomically realistic lung cavity is defined as the radius of a sphere that would have the same surface area as the lung cavity. For both geometries, fluence rate decreases with increasing cavity dimensions, following the expected $1/r^{2}$ dependence, as suggested by Eq. (3).

## 4 Discussion

The comparison among different methodologies across various optical properties reveals important quantitative relationships, as shown in Table 4. For the anatomically realistic lung phantom with a side opening, Monte Carlo simulations demonstrate good agreement with experimental measurements (Tables 4a and 4b), with variations that reflect the complex light-tissue interactions in the anatomically realistic geometry. These experimental validation measurements provide a critical assessment of our computational model’s accuracy under conditions approximating clinical scenarios.

For the spherical cavity model (Table 5a), the theoretical predictions from the modified integrating sphere model [Eq. (3)] show generally good agreement with Monte Carlo simulations, with percentage differences typically within ±10% for most optical property combinations. The agreement tends to be best at moderate optical properties ($\mu_{a}=0.3$ to $0.5\;{\mathrm{cm}}^{-1}$; $\mu_{s}^{\prime }=10$ to $20\;{\mathrm{cm}}^{-1}$), with larger discrepancies observed at extreme values, particularly at low scattering coefficients ($\mu_{s}^{\prime }=5\;{\mathrm{cm}}^{-1}$) where the integrating sphere assumptions become less valid. The diffuse reflectance coefficient ρ in Eq. (3) is calculated using diffusion approximation for a semi-infinite medium (ρ=(a′/2)(1+^−(4/3)A√(3(1−a′))) e−√(3(1−a′))), with transport albedo $a^{\prime }=\mu_{s}^{\prime }/(\mu_{a}+\mu_{s}^{\prime }))$. The equation accounts for refractive index mismatch through the internal reflection parameter $A=(1+r_{a})/(1-r_{a})$, where the Fresnel coefficient $r_{a}$ depends on the relative refractive index $n_{\mathrm{rel}}=n_{\text{tissue}}/n_{\mathrm{me}}d_{\mathrm{ium}}$. Although this provides reasonable approximations for spherical geometries, its accuracy is constrained by diffusion theory assumptions.

For the anatomically realistic lung phantom with a side opening, Monte Carlo simulations demonstrate the best overall agreement with experimental measurements (Table 5b), with a mean absolute difference of 4.6% across all optical properties. The larger deviations observed at $\mu_{a}=0.3\;{\mathrm{cm}}^{-1}$ with high scattering coefficients (Table 5b) warrant additional consideration. Several factors may contribute to these discrepancies. First, the phantom material optical properties, while designed to be minimal, may have a greater relative impact at lower absorption coefficients where the liquid phantom’s absorption is reduced. Second, at high scattering coefficients ($\mu_{s}^{\prime }\geq 30\;{\mathrm{cm}}^{-1}$), achieving complete homogeneity in intralipid-based phantoms becomes increasingly challenging. Studies have shown that high concentrations of intralipid can lead to aggregation and dependent scattering effects^39^ and the addition of stabilizing agents or mixing with solidifying media can destabilize fat emulsions, causing lipid droplets to flocculate or aggregate.^40^ Third, detector spatial averaging effects and positioning uncertainties may have proportionally greater impact when scattered light dominates over direct illumination.^25^ These combined factors likely explain why discrepancies are more pronounced at $\mu_{a}=0.3\;{\mathrm{cm}}^{-1}$ with $\mu_{s}^{\prime }\geq 30\;{\mathrm{cm}}^{-1}$ compared with other optical property combinations. The theoretical model [Eq. (3) with f=0.11] shows larger discrepancies when compared with measurements (Table 5c), particularly at high absorption coefficients where differences can exceed 20%. These discrepancies highlight the limitations of applying simplified analytical models to complex geometries. The comparison between theoretical predictions and Monte Carlo simulations for the lung phantom (Table 5d) reveals a consistent pattern of underestimation at low scattering coefficients and overestimation at high scattering coefficients. This trend suggests that although the modified integrating sphere model provides useful theoretical insights, its limitations in complex geometries further demonstrate why patient-specific Monte Carlo simulations are essential for clinical applications, rather than relying on simplified analytical approximations.

In the spherical cavity model [Fig. 4(a)], boundary fluence rates increase monotonically with increasing scattering coefficient across all absorption values, with $\mu_{a}=0.1\;{\mathrm{cm}}^{-1}$ showing the steepest rise. Conversely, increasing absorption produces a consistent decrease in boundary fluence for all scattering values. The close agreement between simulation and theory validates the integrating sphere approximation for spherical geometries. The anatomically realistic lung model [Fig. 4(b)] demonstrates similar trends but with lower overall fluence rates and increased variability (shown by error bars). For the lung cavity with an air opening [Fig. 4(c)], the overall fluence rates are further reduced compared with the closed cavity models, particularly at high scattering values. The saline-filled lung model with an air opening [Fig. 4(d)] shows slightly higher fluence rates compared with the air-filled model [Fig. 4(c)]. This difference illustrates how the higher refractive index of saline compared with air enhances internal reflection and scattered light contribution despite the presence of an opening.

Previous studies have shown that cavity-like geometries can significantly deviate from simple theoretical predictions, particularly when irregular surfaces, openings, and heterogeneous tissue interfaces are present.^15^^,^^41^ The integrating sphere effect in pleural cavities creates complex light distribution patterns that cannot be accurately predicted by diffusion approximations alone.^42^ Our results demonstrate how geometric complexity can modify these fundamental relationships, with anatomically realistic models showing 49.9% spatial heterogeneity compared with 4.9% in idealized spheres, emphasizing the critical need for Monte Carlo validation in clinical treatment planning.

These optical property relationships provide important clinical guidance, though their interpretation requires an understanding of the underlying physics. Clinical measurements in pleural tissues have shown $\mu_{a}$ values typically ranging from 0.024 to $3.5\;{\mathrm{cm}}^{-1}$ and $\mu_{s}^{\prime }$ values from 1.2 to $40\;{\mathrm{cm}}^{-1}$ during the PDT procedure.^33^^,^^34^ In our study range, tissues with $\mu_{s}^{\prime }>20\;{\mathrm{cm}}^{-1}$ and $\mu_{a}<0.3\;{\mathrm{cm}}^{-1}$ create favourable conditions for light accumulation, potentially requiring reduced treatment times to achieve the prescribed light fluence of $60\;J/{\mathrm{cm}}^{2}$, the established clinical threshold for Photofrin-mediated pleural PDT.^2^ However, although higher fluence rates can reduce treatment time, excessively high fluence rates may lead to rapid oxygen depletion through accelerated photochemical consumption, potentially compromising PDT efficacy despite adequate light delivery.^43^ Thus, comprehensive dosimetry incorporating light distribution, photosensitizer concentration, and tissue oxygenation is required for optimal treatment outcomes.^44^ Conversely, tissues with $\mu_{a}>0.7\;{\mathrm{cm}}^{-1}$ may require extended exposure times or higher source power to compensate for increased photon loss. The relatively uniform scattered light distribution across source positions indicates that multiple-source treatment protocols can maintain consistent background illumination while varying direct light coverage, providing flexibility in treatment planning for irregular cavity geometries.

As demonstrated by Fig. 5, across all geometries, boundary fluence rates increase with increasing scattering coefficient and decrease with increasing absorption coefficient. The spherical model [Fig. 5(a)] shows the highest overall fluence rates, whereas the models with openings [Figs. 5(c) and 5(d)] exhibit the lowest values. The theoretical predictions, which were originally developed for spherical geometries, show excellent agreement with simulation results for the spherical model.

Our finding that source position variations (Fig. 6) showed a negligible effect on scattered light distribution patterns, whereas direct light components remained position-dependent, has important implications for clinical practice. This observation supports current clinical approaches that systematically move the light source throughout the cavity, though it suggests that treatment planning could potentially prioritize certain regions requiring direct illumination.

The size-dependent analysis (Fig. 7) demonstrates that cavity dimensions significantly influence light fluence rate at the cavity boundary, with smaller cavities exhibiting higher fluence rates but potentially greater heterogeneity. This relationship has clinical relevance when treating patients with varying cavity volumes, suggesting that treatment parameters may need adjustment based on cavity size to maintain consistent light dose delivery. The close agreement between our simulation results and theoretical predictions for the spherical model provides validation for the simplified analytical models often used in clinical settings, at least within certain size ranges.

Our findings showing more uniform scattered light distribution in spherical geometries compared with anatomically realistic models indicate that achieving consistent therapeutic doses across irregular pleural surfaces requires careful consideration of cavity geometry. The observed differences between saline-filled and air-filled cavities with openings further highlight the importance of accounting for filling media in treatment planning, as the higher refractive index of saline enhances internal reflection and scattered light contribution.

From a practical standpoint, these findings provide a clear directive for clinical implementation. The 10-fold increase in spatial heterogeneity in anatomical models mandates that patient-specific treatment planning cannot rely on simplified geometric assumptions; instead, it must be based on 3D anatomical data from patient imaging. Planners must explicitly model crucial procedural features, such as the thoracotomy opening, which our results identify as a predictable region of light loss requiring potential dose compensation. Furthermore, our data confirm that pre-treatment assessment of cavity volume and *in vivo* tissue optical properties are essential inputs. This information is not just correlative but predictive, allowing for the adjustment of source power or treatment duration to normalize the delivered dose, thereby mitigating the risk of underdosing in large or highly absorbing cavities and avoiding the excessively high fluence rates that can induce hypoxia and compromise therapeutic efficacy in smaller or highly scattering cavities.

## 5 Conclusion

This study provides a comprehensive characterization of light distribution patterns in pleural PDT through Monte Carlo simulations validated by experimental measurements. Our findings reveal that scattered light distribution is significantly influenced by cavity geometry, tissue optical properties, cavity dimensions, and boundary conditions. The anatomically realistic lung phantom showed more complex behavior with greater spatial variation compared with the idealized spherical model, quantifying the impact of geometric complexity on treatment uniformity. This quantitative framework enables researchers to assess whether simplified geometric approximations are adequate for their specific clinical applications.

These quantitative insights have direct implications for clinical practice and represent a significant advancement in pleural PDT dosimetry. Treatment planning should account for patient-specific anatomical variations, consider cavity size when determining light source power, and potentially compensate for regions near surgical access sites where the scattered light contribution is reduced. The validated computational framework presented here provides a foundation for developing more personalized treatment approaches that can accommodate individual variations in patient anatomy and tissue characteristics. The demonstrated agreement between Monte Carlo simulations and experimental measurements supports the clinical translation of this approach for patient-specific treatment planning. Future efforts should focus on integrating these findings into practical dosimetry tools while incorporating heterogeneous photosensitizer distribution patterns and dynamic changes in tissue optical properties during treatment, ultimately leading to improved treatment consistency, better patient outcomes, and reduced treatment-related complications in pleural PDT.

## Data Availability

Data are available from the authors upon request.
